# Design and Characterization of a Novel Fermented Beverage from Lentil Grains

**DOI:** 10.3390/foods9070893

**Published:** 2020-07-07

**Authors:** Michela Verni, Chiara Demarinis, Carlo Giuseppe Rizzello, Federico Baruzzi

**Affiliations:** 1 Department of Soil, Plant and Food Sciences, University of Bari Aldo Moro, 70126 Bari, Italy; michela.verni@uniba.it (M.V.); carlogiuseppe.rizzello@uniba.it (C.G.R.); 2 Institute of Sciences of Food Production, National Research Council of Italy (CNR-ISPA), 70126 Bari, Italy; demarinischiara@gmail.com

**Keywords:** plant-based milk substitutes, probiotic lactic acid bacteria, legume grain aqueous extracts, antinutritional factors, soluble proteins

## Abstract

The experimental activities carried out in this study aimed at designing a lentil-based beverage rich in soluble and digestible proteins. In order to extract soluble proteins, lentil grains were soaked in water overnight, blended, treated with proteolytic enzymes and fermented with *Lactobacillus* strains. Protein enzymatic hydrolysis, carried out with four commercial food grade enzyme preparations, showed that bromelin, at the enzyme to substrate ratio of 10%, was the best solution to produce this novel beverage. Even though the seven *Lactobacillus* strains were all able to ferment aqueous extract within 24 h, *L. acidophilus* ATCC 4356, *L. fermentum* DSM 20052 and *L. paracasei* subsp. *paracasei* DSM 20312 showed the highest growth rate and the lowest pH values. In fermented lentil-based beverages, the antinutritional factor phytic acid decreased up to 30%, similarly, the highest reduction in raffinose oligosaccharides content reached about 12% the initial concentration. It is worthy of note that the viable density of all strains remained higher than 7 log cfu/mL after 28 days of cold storage. The results here reported show for the first time the possibility to obtain a probiotic lentil-based beverage rich in soluble proteins, peptides and amino acids with low content in main antinutritional factors.

## 1. Introduction

Nowadays, the rising prevalence of milk allergies and lactose intolerance, the shifting to vegan diet, as well as, the awareness of the health and environmental impacts associated with animal derived food products, have influenced consumers towards plant-based drinks as milk alternatives [1,2,3,4]. Plant-based milk substitutes (PBMSs), which can be defined as suspensions of extracted and disintegrated plant material in water, are a fast-growing segment in the new food product category of functional beverages across the globe [2,5]. Indeed, the market for non-dairy milks reached $1.9 billion in 2015, with more than a hundred different variants of PBMSs just in Europe [6].

PBMSs are perceived to be healthy by consumers even though their nutritional value varies considerably depending on the raw material employed. Milk alternatives are commercially obtained from a variety of plants, such as legumes, seeds, nuts, cereals and pseudo-cereals [1,3]. While soya drinks have been largely studied over the years, the amount of research on almond-, rice- and other nut- and seed-based milk substitutes is rather limited. Moreover, the raising interest in these novel products led researchers to focus on their nutritional properties generally considered lower than their animal counterparts [3,7,8]. Vanga and Raghavan [8] recently compared the nutritional content of the most popular PBMSs available on the market. This study concluded that, while soy drinks are currently the best alternative to cow’s milk, almond milk has very low proteins whereas rice and coconut drinks, besides lacking proteins, are also rich in sugar and fat. This unbalance of nutrients poses a health issue; thus, new raw materials and ingredients need to be investigated.

Recently, the emergence of non-dairy probiotic products was pointed out by Min et al. [9] that reviewed the interactions between probiotic microorganisms and several non-dairy food matrices. Authors concluded that even though vegetable foods can be an active carrier for probiotic bacteria, some issues such as microbial cell viability in non-chilled, low pH or high water activity, the occurrence of unwanted fermentation and then the comparison of unacceptable sensory characteristics need to be well evaluated in developing such kind of new foods.

Lentils, whose proteins have been rated equally to soy’s in terms of functional and organoleptic properties [2] are among the matrices recently proposed, alone or as part of a mixture, for producing high value PBMSs [10,11]. Besides for the proper contribution in macronutrients, studies have shown legume-protein based drinks to be similar to cow’s milk in color and viscosity yet deficient in odor and taste [2]. Such beverages also have other nutritional or qualitative shortcomings, anti-nutritional factors such as raffinose-family oligosaccharides or phytic acid, or, most importantly, plant-based proteins may have poor digestibility and often lack some essential amino acids. Lactic acid fermentation has been widely used to improve sensory profile, nutritional properties, texture and microbial safety of PBMSs, avoiding the addition of chemical preservatives (clean label) while preserving their original natural status [12].

*Streptococcus* and *Lactobacillus*, which are able to resist and grow in difficult environments, are the genera mainly employed in plant-based drinks fermentation [13]. Among the *Lactobacillus* spp., *L. casei*, *L. helveticus*, *L. fermentum*, *L. reuteri*, *L. acidophilus*, *L. rhamnosus* and *L. johnsonii* have been extensively used as probiotic cultures in soy-based drinks conferring the broad variety of health benefits associated with their consumption [14]. Interestingly when a kefir beverage was supplemented with germinated and non-germinated wrinkled lentils an improvement into dietary fibers contents, antioxidant activity and calcium solubility was observed [15].

In this context, the use of lentil as an alternative to soy in plant-based beverages was hypothesized. Thus, aiming at formulating a novel fermented lentil-based beverage, in this study a protocol involving proteolytic enzymes and potentially probiotic lactic acid bacteria was optimized. Thus, Lentil–based fermented beverages were evaluated for their biochemical and nutritional composition, and their viable cell count during 28 days of refrigerate storage.

## 2. Materials and Methods

### 2.1. Lentil Grains, Microorganisms and Enzymes

Lentil grains (*Lens esculenta* Moench, agroecotype “Lenticchia di Altamura” safeguarded by the Protected Geographical Indication) purchased from local store and used to set up legume-based fermented beverages had the following proximal composition: moisture 9.0% ± 0.5%, proteins 24.1% ± 0.7% of dry matter (d.m.), lipids, 3.6% ± 0.3% of d.m., carbohydrates 62.5% ± 3.9% of d.m, dietary fibers 17.5% ± 1.2% of d.m., ash 2.2% ± 0.3% of d.m.

*Lactobacillus strains* used in this work (*Lactobacillus acidophilus* ATCC 4356, *Lactobacillus fermentum* DSM 20052, *Lactobacillus gasseri* ITEM 13541, *Lactobacillus helveticus* ATCC 15009, *Lactobacillus johnsonii* NCC533, *Lactobacillus paracasei* subsp. *paracasei* DSM 20312, *Lactobacillus rhamnosus* ATCC 53103) are stored in the Institute of Sciences of Food Production public microbial strain collection. Fresh microbial cultures were routinely propagated in MRS (MRS Broth ISO Formulation, Biolife Italiana Srl, Milan, Italy) for 48 h at 37 °C under anaerobic conditions (ANAEROGEN, AN0025, Oxoid S.p.A., Milan, Italy). When used for fermentation, cultures were centrifuged (10,000 rpm for 3 min) removing the supernatant and resuspending cell pellet into sterile saline solution.

Aiming at maximizing the release of free peptides and amino acid, commercial proteases were used. Papain (60,110 U/mg proteins) and bromelin (2400 U/mg proteins) were purchased from Wisapple Biotech (Beijing, China) whereas Veron HPP (1116 U_Hb_/g, where 1 U_Hb_/g corresponds to the release of 1 mol/min of tyrosine from hemoglobin at 37 °C and pH 5.0) and Veron PS (227 U_Hb_/g) from AB Enzymes GmbH (Darmstadt, Germany).

### 2.2. Setup of the Protocol for Making the Lentil Beverage

#### 2.2.1. Starter Selection

Aiming at selecting the best adapting *Lactobacillus* strain, lentil extracts were prepared. Grains were dipped in tap water for about 16 h; and after hydration, they were drained and weighed. Distilled water was added to the hydrated grains in order to reach the ratio of 1:10 between dry grain and total water. Grain suspensions were homogenized by hand blender, filtered using a gauze; the aqueous extract was sterilized at 110 °C for 10 min. Sterile lentil extracts were singly inoculated with 1% of cell suspension (final density of ca. 6 log cfu/mL) and incubated up to 48 h at 37 °C under anaerobiosis.

Before and after fermentation, the values of pH were determined with a pH-meter (Model 507, Crison, Milan, Italy) with a food penetration probe and presumptive lactic acid bacteria were enumerated using acidified (pH 5.4) MRS (Biolife Italiana) agar medium, supplemented with cycloheximide (0.1 g/L). Plates were incubated under anaerobiosis (AnaeroGen and AnaeroJar, Oxoid) at 37 °C for 48 h. The three best growing lactobacilli were selected for further experiments.

#### 2.2.2. Enzyme Selection

The protocol described by Seo et al. [16], with some modification, was used to select the best performing proteolytic enzyme. Briefly, 20 g of hydrated and blended lentil suspension, prepared as described above, were placed in 50 mL tube and pre-incubated at 30 °C for 1 h, then Papain, bromelin, Veron HPP and Veron PS were added in order to obtain an enzyme/protein ratio of 5% and 10%. More specifically 12.5 mg and 25 mg of commercial preparation of Papain, bromelin, Veron HPP and Veron PS were added to 10 g lentil slurry for 5% and 10% enzyme/protein ratios, respectively. The lentil extract/protease mixtures were vigorously stirred during reaction (200 rpm at 30 °C for 1 h). After the hydrolysis, mixtures were centrifuged (10,000 rpm for 5 min) and filtered using gauze. The enzymes were inactivated at 110 °C for 10 min and then inoculated with different strains.

To assess the degree of hydrolysis for each enzyme, proteins, peptides and amino acids content were determined. The protein concentration was evaluated using the Bradford method [17]. Standard solutions of BSA (bovine serum albumin, Sigma No. A-7030) were used for the calibration line. Subsequently, the absorbance of unknown samples was measured at 595 nm by an automatic spectrofluorometer (Varioskan Flash, Thermo Fisher Scientific, Milan, Italy). The concentration of free peptides and amino acids was measured by the *o*- phthaldialdehyde method [18]. Trifluoracetic acid was added to 2 mL samples to the final concentration of 0.05%. Then the mixture was vortexed (1 min) and centrifuged (10,000 g for 10 min). An aliquot of 25 μL of supernatant was mixed with 975 μL of OPA reagent and immediately employed for absorbance reading at 340 nm in glass couvette. A standard curve of casamino acid mixture (BD bacto-casamino acids, BD Biosciences, San Jose, CA, USA) was used as reference in the range of 0.1 mg/mL–2.0 mg/mL.

### 2.3. Effect of the Thermal Treatment on Survival of Bacillus cereus Endospores

In order to verify the milder thermal treatment able to cut down the microbial load naturally contaminating aqueous extracts, challenge tests were performed using *Bacillus cereus* DSM 4312 as reference strain. *B. cereus* DSM 4312 was cultivated in BHI (Brain Heart Infusion medium, Oxoid S.p.A., Milan, Italy) as previously reported [19]; some drops of this fresh microbial growth were used to inoculate Basal Medium to promote sporulation [20]. Broth culture was diluted to obtain an optical density at 600 nm of 0.3 and then added to the lentil aqueous extracts to about 10^6^ cfu/mL. The inoculated samples were dipped in a water bath and heated at 90 °C for 5 and 10 min or sterilized by autoclave at 110 °C for 5 and 10 min.

Spore count in the inoculated and treated samples was performed on Reinforced Clostridial Medium (RCM, Biolife Italiana), after three days incubation at 30 °C under aerobic conditions.

### 2.4. Manufacture of Lentil Beverages

**Figure 1:**
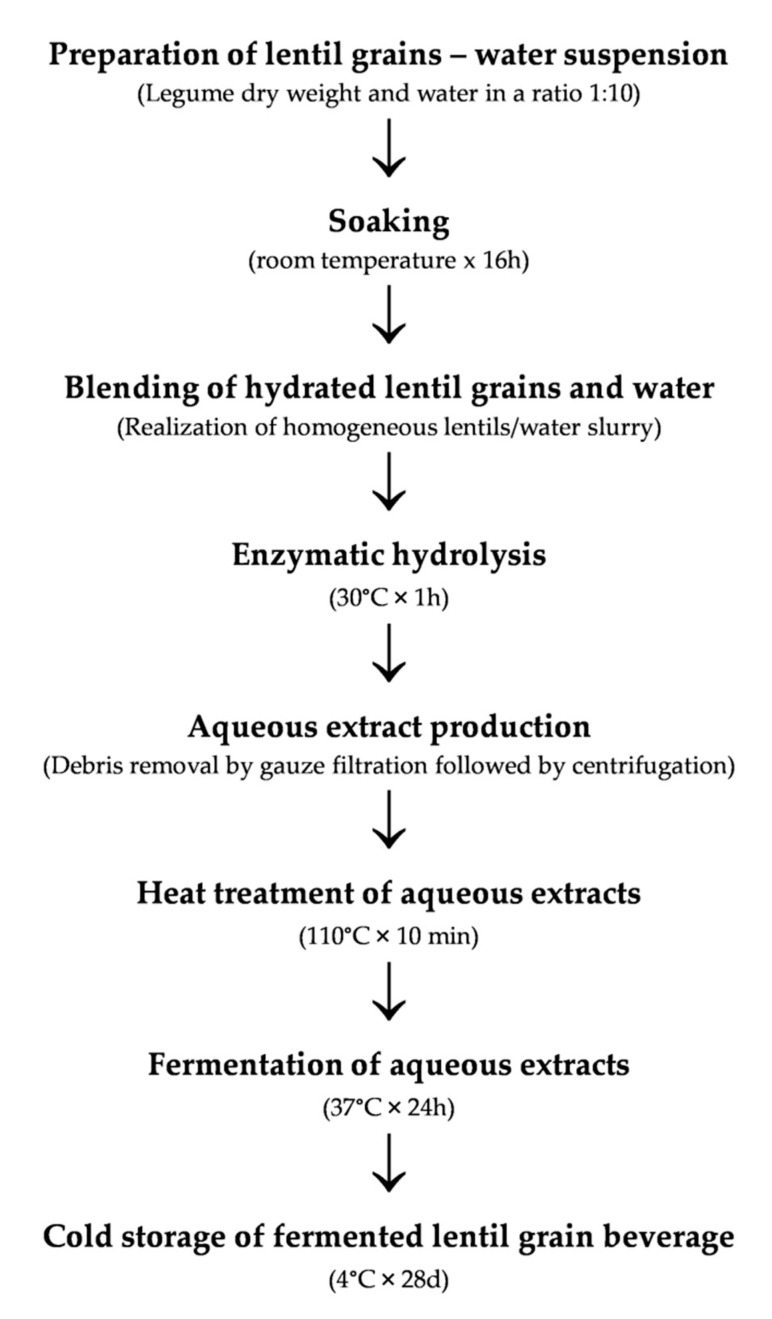
Flow chart for the preparation of a fermented lentil beverage.

Based on the results obtained during the enzyme and starter selection, a protocol for the lentil beverage was setup (Figure 1).

Lentil grains were washed, then dipped in tap water (ratio 1:10) and incubated at room temperature for 16 h. After hydration, soaked lentils were hand-blended to obtain a homogeneous slurry that was subjected to enzymatic hydrolysis under the best conditions previously defined (Section 2.2.2). After 1 h at 30 °C, the mixture was cotton-gauze filtered for debris removal, centrifuged (10,000 rpm per 5 min) and treated at 110 °C for 10 min. Sterile lentil grains extracts were singularly inoculated (final density of ca. 6 log cfu/mL) with *L. acidophilus* ATCC 4356 (LeA) or *L. fermentum* DSM 20052 (LeF) or *L. paracasei* subsp. paracasei DSM 20312 (LeP) and incubated for 24 h at 37 °C under anaerobiosis. A lentil beverage (Le) not inoculated was used as control. Before and after fermentation, pH and presumptive lactic acid bacteria were determined as described above.

### 2.5. Biochemical, Nutritional and Microbial Characterization of the Beverages

#### 2.5.1. Organic Acids, Sugars, Peptides and Amino Acids

Lactic and acetic acids contained in the beverages were determined by High Performance Liquid Chromatography (HPLC), using an ÄKTA Purifier system (GE Healthcare, Buckinghamshire, UK) equipped with an Aminex HPX-87H column (ion exclusion, BioRad, Richmond, CA, USA), an UV detector operating at 210 nm and a refractive index detector (PerkinElmer 200a) operating at 60 °C. H_2_SO_4_ 10-mmol/L was used as mobile phase with a flow rate of 0.6 mL/min [21]. The fermentation quotient (FQ) was determined as the molar ratio between lactic and acetic acids.

Carbohydrates (fructose, glucose, sucrose and the α-galactosides) were determined by HPLC as described in Hernandez et al. [22]. The chromatographic system was composed by 1200 quaternary pump, 1260 column compartment, 1260 refractive index detector and manual Rheodyne injector with 10 µL loop (Agilent, Santa Clara, CA, USA). A Spherisorb Amino (NH2) column 8 nm, 5 µm, 4.6 mm × 250 mm (Waters, Milford, MA, USA) was used for separation under isocratic conditions at 28 °C. The temperature of the detector was set at 40 °C and data acquisition and processing were performed by Chemstation v. 2.0 (Agilent). Organic acids and carbohydrates, used as standards were purchased from Sigma Chemical Co. (Milan, Italy).

The concentration of proteins and peptides was determined on the beverages, to evaluate the degree of proteolysis as described in Section 2.2.2. Total free amino acids were analyzed by a Biochrom 30 series Amino Acid Analyzer (Biochrom, Ltd., Cambridge Science Park, England) with a Na-cation-exchange column as described by Rizzello et al. [21].

#### 2.5.2. Dietary Fibers and Resistant Starch

Total dietary fibers (TDF) content was determined on the beverages as described by Goni et al. [23]. Briefly, freeze-dried samples were subject to a first digestion with pepsin solution, 1 h at 40 °C, then with pancreatin at 37 °C for 6 h. After centrifugation, the supernatants were removed and further digested with amyloglucosidases and dialyzed to determine soluble dietary fibers (SDF), whereas the pellets were dried overnight, cooled in a desiccator and weighed to determine insoluble dietary fibers (IDF).

Resistant starch (RS) and phytic acid content were determined on freeze-dried beverages by using the Resistant Starch K-RSTAR and K-PHYT 05/07 (Megazyme Int., Bray, Ireland), respectively, following the manufacturer’s instructions.

#### 2.5.3. Antioxidant Activity

The concentration of total phenols was determined as described by Slinkard and Singleton [24] using Folin–Ciocâlteu reagent (Sigma Chemical Co.) and expressed as gallic acid equivalent. The scavenging activity on 1,1-diphenyl-2-picrylhydrazyl (DPPH) free radical was measured according to the method of Yu et al. [25] with some modifications. Freeze-dried beverages were dissolved in 0.1-M phosphate buffer at pH 7.0, at the final concentration of 1 mg/mL of peptides, and 2 mL of each solution were added to 2 mL of 0.1-mM DPPH dissolved in 95% ethanol. The mixture was shaken and left for 30 min at room temperature, and the absorbance of the resulting solution was read at 517 nm. Antioxidant activity was expressed as follows:

$$\mathrm{DPPH}\;\mathrm{scavenging}\;\mathrm{activity}\;(\%)=100\times \frac{\left(\mathrm{blank}\;\mathrm{absorbance}-\mathrm{sample}\;\mathrm{absorbance}\right)}{\mathrm{blank}\;\mathrm{absorbance}}$$

The value of absorbance was compared with 75 ppm butylated hydroxytoluene (BHT), which was used as the antioxidant reference.

#### 2.5.4. Shelf Life Evaluation

Changes in microbial populations and acidification activities of the beverages were evaluated by plate counts and pH measurements performed each 7 days for the cold storage (4 °C) period of 28 days.

### 2.6. Statistical Analysis

The results were calculated as means of at least three replicates. The data were analyzed by one-way ANOVA; pair-comparison of treatment means was obtained by Tukey’s procedure at *p* < 0.05, using the statistical software Statistica 8.0 (StatSoft, Inc., Tulsa, OK, USA). Significantly different data were indicated with a different superscript letter.

## 3. Results

### 3.1. Setup of the Bioprocessing Protocol

Before fermentation, lentil extracts had a pH of 6.83 ± 0.07. Even though all strains confirmed the ability to grow and acidify the medium, only *L. acidophilus*, *L. fermentum* and *L. paracasei* increased their viable amount more than 2 log cfu/mL within 24 h and for this reason were chosen for further assays (Table 1).

Aiming at increasing the release of free peptides and amino acids, four food grade proteolytic enzymes were screened. Lentil extract obtained without the hydrolysis, had a protein content of 23.48 ± 0.56 mg/mL, which decreased after each enzymatic treatment. The concentration of proteins was lower than 0.50 mg/mL after treatment with bromelin, Papain and Veron HPP and higher than 3 mg/mL with Veron PS; in all cases the increase of the enzyme concentration from 5%–10% produced final lower protein content. As the protein content decreased, free peptides and amino acids increased. Lentil extracts hydrolyzed with Veron PS had peptides and amino acids 40% and 50% higher the than the control (0.36 ± 0.05 mg/mL), when used at 5%–10%, respectively. When the other enzymes were used, the initial concentration of peptides and amino acids increased up to 4-fold, except for bromelin (10%) which released up to 2.4 ± 0.26 mg/mL (almost 7-fold higher).

Since the lentil extract carried several microorganisms potentially able to ferment or spoil it (data not shown), a challenge test, aiming at defining the milder thermal treatment sufficient to stabilize the beverages, was carried out with the food poisoning *Bacillus cereus* DSM 4312. Lentil extracts containing spore suspensions (10^6^ spores/mL) were heated at 90 °C and 110 °C. The treatment at 90 °C for 5 and 10 min revealed the presence of colonies (about 3 log cfu/mL) as well as that at 110 °C for 5 min, whereas only in samples treated at 110 °C for 10 min no colony was found. In addition, microbial growth was not observed when the same sample was incubated for 24 h at 37 °C.

### 3.2. Biochemical and Nutritional Characterization of the Beverages

Prior to fermentation, the beverage pH was 6.42 ± 0.06 and decreases up to 5.03 were detected after the incubation with the three starter strains previously defined. After 24 h at 37 °C their microbial cell density increased, of ca. 2 log cycles, compared to the initial inoculum level, with the highest value recorded for *L. fermentum* at 7.87 log cfu/mL.

Lactic and acetic acids were detected in traces before fermentation whereas in fermented sample ranged from 6.21 ± 0.36 to 9.68 ± 0.62-mM and from 0.77 ± 0.06 to 1.25 ± 0.09 mM, respectively. Compared to the beverages fermented with *L. paracasei*, significantly (*p* < 0.05) higher lactic acid content was found in those extracts fermented with *L. acidophilus* and *L. fermentum*. Indeed, the quotient of fermentation (molar ratio between lactic and acetic acids, QF) was 10.69 ± 0.59, 9.63 ± 0.42 and 6.58 ± 0.27 in LeA, LeF and LeP, respectively. Ethanol was not found.

As shown in Table 1, stachyose was the most abundant sugar in unfermented lentil extracts followed by sucrose. After fermentation, glucose was not detected in any of the fermented beverages. Except for LeP, verbascose completely disappeared in LeA and reduced of ca. 70% in LeF. However, with high variability among samples, significantly (*p* < 0.05) lower amount of stachyose, raffinose and fructose were detected in all fermented beverages. The beverage fermented with *L. paracasei*, was the one showing the lowest reduction for all sugars (Table 2).

As a result of fermentation, a feeble proteolysis was observed. Compared to the unfermented beverage (21.67 ± 1.41 and 4.96 ± 0.14 mg/mL of proteins and peptides, respectively), decreases in both concentrations were detected. Protein content decreased from 25%–50%, whereas peptide from 40% to 45%, with LeA and LeP having the lowest and highest reduction, respectively.

**Figure 2:**
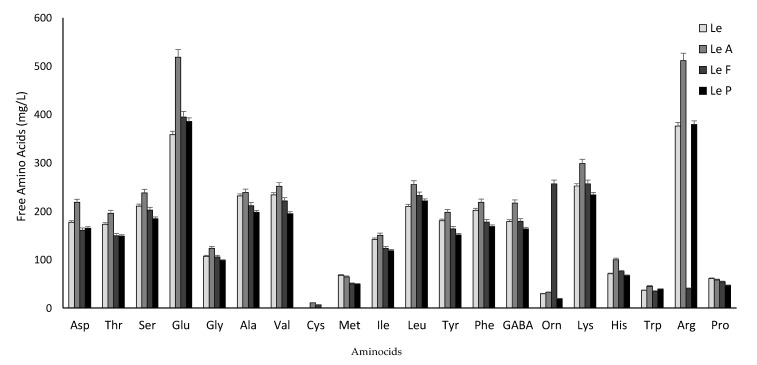
Concentration of free amino acids (mg/L) of beverages made with hydrolyzed lentil grains and fermented with *Lactobacillus acidophilus* ATCC 4356 (LeA), *L. fermentum* DSM 20052 (LeF), *L. paracasei* subsp. *paracasei* DSM 20312 (LeP) for 24 h at 37 °C. A beverage without bacterial inoculum (Le) was used as control. Data are the means of three independent experiments twice analyzed. Bars of standard deviations are also represented.

The concentration of free amino acids (FAA) of the beverages is shown in Figure 2. Compared to Le (3360 ± 0.11 mg/L), total FAA were slightly, but significantly (*P* < 0.05) higher in LeA (ca. 4000 mg/L) whereas it did not change in LeF and LeP. Except for Pro and Orn, during fermentation, the concentration of all the FAA increased in LeA. Considerably, higher amounts of Glu and Arg were found in comparison with Le, whereas Cys was only detected in LeA and LeF. Substantial decrease of Arg and increase of Orn were found in beverages fermented with *L. fermentum*.

In order to determine the effect of fermentation on phytic acid, its content was also measured before and after incubation. *L. acidophilus* and *L. fermentum* were able to decrease phytic acid up to 30% the initial concentration (30.90 ± 1.45 μg/mL). On the contrary, no significant (*P* > 0.05) changes were detected in the beverage fermented with *L. paracasei*.

### 3.3. Total Phenols and Antioxidant Activity

However, slightly higher in LeF and LeP, the concentration of total phenolic compounds in the beverages (ca. 3.3 mM) did not differ significantly (*P* > 0.05) before and after fermentation. On the contrary, the scavenging activity on DPPH radical was largely affected by fermentation. The activity of all beverages was lower than that of BHT (ca. 80%), used as the positive control, yet up to 11-fold higher than the unfermented beverage which had antioxidant activity lower than 4%. LeA showed the highest activity (40.73% ± 2.05%), followed by LeP and LeF (28.61% ± 1.27% and 31.74% ± 1.45%, respectively).

### 3.4. Dietary Fibers and Resistant Starch

Total dietary fibers of the control beverage were 2.78 ± 0.12 g/L, of which 98% were insoluble. The concentration of total dietary fiber did not significantly (*P* > 0.05) differ among the beverages before and after incubation. Lactic acid fermentation caused a significant (*P* < 0.05) increase of the soluble fraction. In particular, the increase varied from 8- to 20-fold, with LeF and LeP showing the highest content (0.98 ± 0.07 and 0.79 ± 0.05 g/L, respectively). As expected, an opposite trend was found for insoluble dietary fibers which were 2.31 ± 0.19, 1.78 ± 0.14 and 2.01 ± 0.16 g/Lin LeA, LeF and LeP, respectively. The control beverage contained 0.18 ± 0.01% of RS which slightly yet significantly (*P* < 0.05) increased during fermentation, especially with *L. fermentum*, reaching up to 0.25%.

### 3.5. Shelf life of Beverages

As the viability of lactic acid bacteria during cold storage is concerned, it is possible to state that at the end of incubation the microbial cell density of all strains increased, of about two logarithmic cycle in comparison with initial concentration. The values of viable bacterial populations in legume grain watery extracts during 28 days of cold storage are shown in Table 3.

The lactobacilli chosen for this work, even though isolated from other sources, were found to be able to growth into a new food matrix and to survive also in them during one month of cold storage. pH value did not change during cold storage remaining stable at 4.89 ± 0.67 depending on the strain and the extension of cold refrigerate period.

## 4. Discussion

The increasing interest towards plant-based beverages, driven by the expansion of the market for food intolerance products, as well as for functional foods, has led scientific community to explore new matrices and bioprocessing protocol to provide a balanced profile [1,2,3]. Generally, water is used to extract the plant material, the addition of other ingredients, as well as several processing steps, such as heat treatments, fermentation and/or homogenization, are usually considered to prepare and formulate the final product [26]. In this study, three steps were optimized in order to set up the bioprocessing protocol for a lentil-based drink (Figure 1): enzymatic proteins hydrolysis from lentils slurry, heat treatment and fermentation with selected lactic acid bacteria.

Like most legumes, lentil is a source of protein, mainly globulins, with concentration varying between 21% and 32%. Since their extraction is highly dependent on pH, temperature and solvent ratio [27], to maximize their release, a hydrolysis with four food grade proteases was included in the protocol and evaluated. bromelin at 10%, which led to a release in peptides and amino acids, almost 7-fold higher than that in Le, was selected.

Minimally processed chilled food, like PBMSs, if not properly treated, pose a risk to human health because of *Bacillus cereus,* a psychotropic food poisoning bacterium. Therefore, a challenge test, aiming at defining the milder thermal treatment sufficient to stabilize the beverages, was carried out. Despite 2 min at 90 °C is the average time necessary to the first 10-fold reduction in spore concentration [28] test revealed that the heat treatment, able to deactivate the enzyme and sterilize the beverages before fermentation, had to be carried out at 110 °C for 10 min.

Finally, seven lactic acid bacteria, chosen among species commonly known for their probiotic properties [14], were screened for the fermentation step. In accordance with the literature, even though isolated from other sources, all the lactobacilli were found to be able to growth into a new substrate, demonstrating how vegetable matrices can be fermented by using both autochthonous lactic acid bacteria [29] or selected *Lactobacillus* strains [30]. The strains selection, based on the highest grow rate within 24 h, allowed to identify *L. acidophilus* ATCC 4356, *L. fermentum* DSM 20052 and *L. paracasei* subsp. *paracasei* DSM 20312. These strains were also able to survive during cold storage; in addition, despite the high vital concentration found for all strains (ca. 8 log cfu/mL), no significant post-acidification phenomenon was found. The absence of additional acidification of food matrix during cold storage is considered useful to preserve microbial viability of foods carrying living probiotic strains and the original sensory characteristics [31,32]. In addition, it is well known that the minimum concentration of probiotics necessary to achieve a specific therapeutic benefit is strain dependent, the viable load of all lactobacilli at the end of cold storage was found higher than 6 log cfu/mL, usually considered the lower cellular density useful to play a positive role in human health [32].

Fermentation in conjunction with enzymatic treatments was used as a tool for sugar reduction in a quinoa-based drinks, lowering glucose content by 40% and the glycemic load by 35% compared to the untreated control [5]. Legumes also contain variable concentration of α-galactosides of sucrose (RFOs), namely raffinose, stachyose and verbascose [12]. While moderate doses favor the metabolism of beneficial intestinal microorganisms (e.g., *Bifidobacteria*), due to the lack of α-galactosidase activity, RFOs are not degraded in the upper gastrointestinal tract, thus causing gastrointestinal symptoms [33].

In the condition of this study, as already reported in soy drinks fermented with *L. acidophilus* [34], *L. fermentum* [35] and *L. paracasei* [36], fermented beverage had lower (from 13% to 75%) RFOs content, when compared with the unfermented control. It is worthy of note that the total RFOs content of unfermented beverage was reduced at less than 200 mg/L after fermentation with *L. acidophilus* ATCC 4356.

One of the risks of replacing cow’s milk with PBMSs, especially for young children, is the extremely low protein content of some products, which is why the raw material and the processing technology adopted play a crucial role [1]. All legume storage proteins are relatively poor of sulfur-containing amino acids (Met, Cys) and Trp [37] and, in this study, only *L. acidophilus* fermentation caused a marked increase, up to 18%, of the total FAA concentration (e.g., Asp, Glu, Leu, Lys, Arg). In LeF and LeP, on the contrary, a slight reduction was observed, probably because the amino acids were used for the growth of the strains. However, Cys that was not detected in Le, reached 11 mg/L and γ-aminobutyric acid (GABA), a nonprotein amino acid inhibitory neurotransmitter in the brain responsible for regulating blood pressure [38], increased of 20% in LeA (Figure 2). The detection of GABA could be the combined result of the soaking and the fermentation steps as already demonstrated in for adzuki beans in which the GABA content increased 150-fold after preprocessing and 20-fold after fermentation in comparison with the non-treated or not-fermented adzuki beans, respectively [38]. Since lentil beverages contained up to 200 mg/L of GABA, specific in vivo trials should be carried out in order to verify if the beneficial anti-fatigue effect claimed by Kanehira et al. [39] produced by 50 mg GABA oral intake or the anti-stress effect recorded by Yoto et al. [40] after ingesting capsules containing 100 mg of GABA can be also obtained by drinking the corresponding amount of lentil fermented beverage.

Proteinase and peptidase activities of lactic acid bacteria may also release peptides and free amino acids with strong antioxidant activity [41]. However, antioxidant activity of foods has been bestowed to phenolic compounds for decades, examples of bioactive peptides in fermented legumes have been reported [41,42]. Explaining why, in the conditions of this study, despite the total phenolic content did not change during fermentation, a considerable increase in the antioxidant activity on DPPH radical of the beverages was observed.

Bioavailability of minerals such as Ca^++^, Mg^++^, Fe^++^ and Zn^++^ can be reduced by phytic acid, an anti-nutritional factor largely occurring in many legume flours [10]. As this product was thermally treated prior fermentation, which would destroy endogenous phytases, but not the heat-stable phytic acid, its decrease in LeA was ascribed to fermentation. This result is in accordance with that obtained by Tang et al. [43] who observed phytase activity and consequent phytic acid reduction in a soy beverage fermented with *L. acidophilus*.

Prebiotics are non-digestible food components that are specifically utilized by probiotics also in the lower gastrointestinal tract [44]; in particular resistant starch represents a small fraction of starch that is resistant to hydrolysis by digestive enzymes in vitro and in vivo, and it is considered part of the dietary fiber fraction thanks to its functional properties [45]. The bioprocessing technology developed in this study allowed an increase of resistant starch up to 25%, which is partially due to the acidification caused by the organic acids released during fermentation [46]. The daily consumption of foods rich in dietary fiber (e.g., legumes) is recommended to prevent several diseases, to regulate energy intake and satiety and to help the glycemic control in diabetic patients [37]. In this study, the content of total dietary fiber did not show significant variations before and after fermentation. However, the ratio between insoluble (lignin, cellulose and some hemicelluloses) and soluble (pectin, some hemicelluloses and other non-starch polysaccharides) dietary fibers decreased after fermentation (up to 50%), as previously reported in cereal-based beverages [47].

It can be concluded that, by increasing the potential pro- and prebiotic of the drink, while increasing amino acid content, the protocol optimized in this study showed adequate potential for industrial applications, even though more in-depth analyses (e.g., sensory evaluation or the possible release of toxic biogenic amines) still need to be carried out. In addition, the application of different and innovative food technologies could simplify and improve the efficiency of the laboratory protocol here set up for the realization of several fermented beverage from different legumes.

## Figures and Tables

**Table 1:** Changes in cell density and pH values of lentil watery extracts fermented with different *Lactobacillus* strains after incubation at 37 °C for 24 and 48 h.

Strain	T24		T48	
	Δlog	ΔpH	Δlog	ΔpH
*L. acidophilus* ATCC 4356	2.71	−0.66	3.63	−1.64
*L. fermentum* DSM 20052	3.13	−1.26	3.20	−1.40
*L. gasseri* ITEM 13541	1.57	−1.60	2.40	−1.91
*L. helveticus* ATCC 15009	1.32	−1.20	2.30	−1.21
*L. johnsonii* NCC533	1.57	−1.80	1.98	−2.41
*L. paracasei* DSM 20312	2.01	−0.52	2.82	−0.77
*L. rhamnosus* ATCC 53103	1.47	−1.58	1.86	−2.01

**Table 2:** Sugars concentration (mg/L) in beverages made with hydrolyzed lentil grains and fermented with *Lactobacillus acidophilus* ATCC 4356 (LeA), *L. fermentum* DSM 20052 (LeF), *L. paracasei* subsp. *paracasei* DSM 20312 (LeP) for 24 h at 37 °C. A beverage without bacterial inoculum (Le) was used as control.

Sugar	Le	LeA	LeF	LeP
**Fructose**	70.95 ± 2.69 ^a^	21.26 ± 0.99 ^c^	39.56 ± 1.67 ^b^	35.75 ± 1.78 ^b^
**Glucose**	118.36 ± 4.89	nd	nd	nd
**Sucrose**	984.87 ± 30.55 ^a^	439.80 ± 14.33 ^c^	298.45 ± 7.95 ^d^	598.70 ± 17.93 ^b^
**Raffinose**	224.83 ± 10.16 ^c^	147.08 ± 5.62 ^d^	333.88 ± 14.85 ^b^	693.66 ± 33.28 ^a^
**Stachyose**	1309.59 ± 63.68 ^a^	35.96 ± 1.09 ^d^	421.72 ± 19.23 ^c^	849.20 ± 38.26 ^b^
**Verbascose**	33.72 ± 1.29 ^a^	nd	10.54 ± 0.46 ^b^	32.99 ± 1.42 ^a^

The data are the means of three independent experiments ± standard deviations (*n* = 3). ^a–d^ Values in the same row, with different superscript letters, differ significantly (*P* < 0.05). nd: not detected.

**Table 3:** Cell density, in log cfu/mL of lactobacilli, in lentil watery extracts during 28 days of cold storage.

Day of 4 °C storage	*L. acidophilus* ATCC 4356	*L. fermentum* DSM 20052	*L. paracasei* DSM 20312
0	7.70 ± 0.20	7.87 ± 0.12	7.33 ± 0.11
7	8.22 ± 0.23	8.12 ± 0.25	8.01 ± 0.48
14	8.18 ± 0.28	7.72 ± 0.44	8.04 ± 0.41
21	7.29 ± 0.31	7.38 ± 0.19	8.22 ± 0.28
28	7.50 ± 0.12	7.20 ± 0.24	8.03 ± 0.17
